# Physiological adaptations of *Saccharomyces cerevisiae* evolved for improved butanol tolerance

**DOI:** 10.1186/1754-6834-6-101

**Published:** 2013-07-15

**Authors:** Payam Ghiaci, Joakim Norbeck, Christer Larsson

**Affiliations:** 1 Department of Chemical and Biological Engineering, System and Synthetic Biology, Chalmers University of Technology, Gothenburg, Sweden

**Keywords:** *Saccharomyces cerevisiae*, Butanol, Tolerance, Proteomics

## Abstract

**Background:**

Butanol is a chemical with potential uses as biofuel and solvent, which can be produced by microbial fermentation. However, the end product toxicity is one of the main obstacles for developing the production process irrespective of the choice of production organism. The long-term goal of the present project is to produce 2-butanol in *Saccharomyces cerevisiae*. Therefore, unraveling the toxicity mechanisms of solvents such as butanol and understanding the mechanisms by which tolerant strains of *S. cerevisiae* adapt to them would be an important contribution to the development of a bio-based butanol production process.

**Results:**

A butanol tolerant *S. cerevisiae* was achieved through a series of sequential batch cultures with gradual increase of 2-butanol concentration. The final mutant (*JBA-mut*) tolerates all different alcohols tested at higher concentrations compared to the wild type (*JBA-wt*). Proteomics analysis of the two strains grown under mild butanol-stress revealed 46 proteins changing their expression by more than 1.5-fold in *JBA-mut*, 34 of which were upregulated. Strikingly, 21 out of the 34 upregulated proteins were predicted constituents of mitochondria. Among the non-mitochondrial up-regulated proteins, the minor isoform of Glycerol-3-phosphatase (Gpp2) was the most notable, since it was the only tested protein whose overexpression was found to confer butanol tolerance.

**Conclusion:**

The study demonstrates several differences between the butanol tolerant mutant and the wild type. Upregulation of proteins involved in the mitochondrial ATP synthesizing machinery constituents and glycerol biosynthesis seem to be beneficial for a successful adaptation of yeast cells to butanol stress.

## Background

There is a necessity of finding substitutes for finite fossil resources in production of fuels as well as various chemicals that nowadays are obtained as petroleum derivatives. Butanol is a class of chemical products which has several applications such as fuel, industrial solvent etc. [1] and it has several advantages in comparison with ethanol as a biofuel, such as higher energy density, lower water content and vapor pressure [1-3]. It has mostly been produced as a petrochemical but there are also biological alternatives available [4,5]. Both native and engineered microorganisms have been used as butanol producers [1,6-11]. Some species of *Clostridium* genus (e.g. *C. acetobutylicum* and *C. beijerinckii*) are known to produce 1-butanol naturally, mixed with acetone and ethanol [6]. However, the limited availability of genetic tools makes *Clostridia* less competitive compared to species like *Escherichia coli* and *S. cerevisiae* where genetic manipulation techniques are well developed with regards to successful heterologous protein expression. *S. cerevisiae* in particular is a well-studied organism with a long history of industrial use; e.g. in brewing and ethanol production. Several groups have engineered *S. cerevisiae* to produce 1-butanol and isobutanol either through redirecting amino acid biosynthetic pathways or by introducing the 1-butanol pathway of *C. acetobutylicum*[12,13]. The same strategies have been applied to produce 1-butanol and isobutanol in *E. coli*[8-10]. However, for all production organisms, natural or engineered, end product toxicity is one of the main limiting factors in developing an effective butanol production process [14], i.e. the tolerance level towards butanol is rather similar for *Clostridia* and *S. cerevisiae*[14-16]. Different isomers of butanol exhibit different extents of toxicity. 1-butanol being the most toxic with concentrations above 1.5-2% (v/v) being inhibitory for most cells including the native 1-butanol producers [14,16,17] while iso-butanol and 2-butanol can be tolerated at higher levels [12,18]. One of the reasons for this is probably that 1-butanol is the most hydrophobic molecule with the strongest ability to permeate and/or interact with the cellular membrane [19-26]. Different alcohol toxicity mechanisms have been described such as accumulation of toxic metabolites [27], changes in membrane fluidity and transport disturbance [19,20,22-26,28-30] as well as disorders in translation initiation [31].

In this study a strain of *S. cerevisiae* was evolved to become more tolerant towards 2-butanol by making sequential batch cultures with increasing 2-butanol concentrations. However, the resulting strain was also more tolerant to all alcohols tested. In order to understand the mechanisms that are important for the observed butanol tolerance we compared the protein expression profile, lipid content and growth behavior of the evolved tolerant mutant (*JBA-mut*) and wild-type (*JBA-wt*). Results from these studies point to the importance of mitochondria and glycerol synthesis as tolerance determinants.

## Results and discussions

### Strain evolution

**Figure 1:**
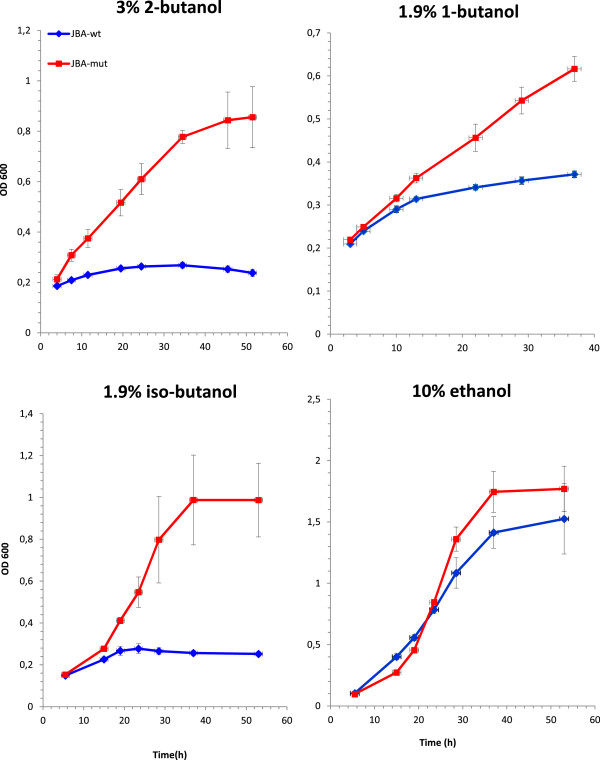
**Growth of *JBA-wt *(blue open squares) and *JBA-mut *(red filled squares) in YPD media supplemented with 3% (v/v) 2-butanol, 1.9% (v/v) 1-butanol, 1.9% (v/v) iso-butanol and 10% (v/v) ethanol, respectively.** Three independent sets of cultivations were performed for each strain and error bars indicate the standard deviations.

A butanol tolerant yeast (*S. cerevisiae*) was selected by evolution under butanol stress. The starting yeast strain (*JBA-wt*) which is inherently quite ethanol tolerant [32] was cultivated in sequential batches while exposed to stepwise increases in concentrations of 2-butanol (1.9%, 2.5% and 3% v/v). Transfer of the strain to a fresh media with higher 2-butanol concentration was done provided that growth rate in the previous concentration had been constant for several transfers. After a total of 30 serial transfers (within 24 days and equivalent to roughly 100 generations) the cells could grow in 3% v/v 2-butanol while the wild type strain (*JBA-wt*) could not sustain growth at this concentration (Figure 1). Our selection pressure was exposure to 2-butanol, but we were also interested in investigating whether this strain would display an increased tolerance to the other isomers of butanol as well as ethanol and propanol, or whether this ability is exclusive for 2-butanol. The results showed that the evolved strain was more resistant to both 1-butanol and iso-butanol (Figure 1). In addition, the ethanol tolerance was improved although not to the same extent as for butanol (Figure 1). The tolerance to 1-propanol was also increased (data not shown). Hence, we conclude that the *JBA mut* strain displays an increased tolerance especially to inhibitory concentrations of higher alcohols and hence might serve as a suitable model to elucidate the cellular adaptation mechanisms leading to alcohol tolerance.

### Protein expression profile

With the evolved tolerant strain (*JBA-mut*) in hand, it was of obvious interest to investigate into the molecular mechanisms underlying the tolerance phenotype. Different approaches might be chosen to assess physiological adaptations of an evolved strain. In this study we chose to look at the differences on the level of the proteome, lipid composition and physiological properties. Applying a SDS-associated protein extraction coupled to protein trypsin digestion and mass spectrometry (MS) analyses allowed us to also study poorly soluble proteins (e.g. membrane bound proteins). This was important since butanol stress is likely to act via changes in plasma membrane properties, possibly involving changes in the expression of membrane associated proteins, which are known to be difficult to quantify.

Many stresses, including exposure to butanol, lead to a reduced growth rate, which is in turn a major source of gene expression changes [33]. In order to determine a threshold value at which the mutant (*JBA-mut*) and the wild type (*JBA-wt*) strain exhibited a similar growth rate we performed a screen of the growth rate of our mutant strain and the corresponding wild type strain in a range of 2-butanol concentrations. The highest concentration of 2-butanol at which the growth rates of the two strains was identical was determined to be approximately 1.2% (v/v), which was therefore chosen as the condition for cultivation and harvest of cells for protein extraction and MS analysis. At this concentration of 2-butanol, an approximately 10% reduction in growth rate was seen for both strains, indicative of cellular stress by 2-butanol. Cells were grown in biological triplicates in 1.2% (v/v) 2-butanol and harvested at OD_600_ ≈ 1 (corresponding to roughly 1 x 10^7^ cells/ml), a point at which exponential growth would still continue for about two generations and nutrient limitation would not be a major problem. Following the harvest, proteins were extracted, digested with trypsin and labeled with TMT-reagents (as described in the materials and method section). In the subsequent LC-coupled mass spectrometric analysis, we were able to quantify approximately 1100 proteins from the *JBA-wt* and *JBA-mut* strains. These 1100 proteins contained representatives from essentially all cellular locations (notably also many plasma membrane proteins), which we take as verification for the utility of our choice of extraction and trypsination method. Most components of the central metabolic pathways were quantified, as well as essentially all components of the ribosomes, together with many representatives of structural and regulatory factors. It is likely that many more proteins of lower abundance could have been quantified in a second round of targeted mass spectrometric analysis, by actively avoiding the peptides analyzed in the first round. However, given the rather clear conclusion from the primary analysis of protein expression (see below) we decided to not go on with such an analysis.

The expression of ribosomal proteins are usually good indicators of changes in growth rate [33] and we therefore first looked at the expression of these proteins. In good agreement with our choice of culture conditions giving a similar growth rate for both strains, it was found that the expression of ribosomal proteins did not change significantly (an average mutant/wt expression change of 0.93 +/− 0.06 for 40S-subunits and 0.92 +/− 0.12 for the 60S-subunits). This result indicates that any changes in protein expression would most likely not be caused by differences in growth rate.

We next decided to narrow down the data on quantification of ~1100 proteins by a Student’s t-test (using a ≥95% significance threshold). 34 proteins were found with at least 1.5 fold up-regulations (Table 1) while 12 proteins were down-regulated 1.5 fold or more by the same criteria (Table 2) when comparing the *JBA-mut* to the *JBA-wt* strain. Most strikingly, 21 out of 34 up-regulated proteins were mitochondrially associated, among which 12 were components of respiratory chain including ATP-synthase and cytochrome c1 which is clearly a highly significant enrichment. The fact that about two-thirds of the upregulated proteins were mitochondrial is a strong indication of higher mitochondrial activity in *JBA-mut*. This might be explained as an effort to compensate high ATP consumption rates due to malfunction of the plasma membrane barrier or poor performance of ATP synthesis. Among the non-mitochondrial proteins most upregulated in the *JBA-mut* strain we found Lactoylglutathione lyase (Glo1), the small heat shock protein (Hsp42), the osmotically regulated glycerol 3-phosphatase (Hor2/Gpp2) and Glucokinase I (Glk1). Hor2/Gpp2 is one of two highly similar isogenes coding for the last step in glycerol biosynthesis [34] and it is also part of the general stress response [33]. The upregulation of Hor2/Gpp2 is interesting in view of the fact that upregulation of the highly similar isogene *RHR2/GPP1* was found to be a strain specific determinant (SSG) of ethanol tolerance [35], suggested to mediate an increased capacity for NADH oxidation. A need for NADH oxidation would also be compatible with the observed increase in expression of proteins in the mitochondrial respiratory chain in the *JBA-mut* strain. However, similar to the study by Stanley et al. [35], we do not observe any significant change in any of the other glycerol metabolic enzymes, even though a slightly increased yield of glycerol was found (see below). Glo1 is involved in the detoxification of methylglyoxal the increased production of which is commonly seen under many forms of stress, notably during conditions of increased glycerol production [36]. The expression of glucokinase I (Glk1) is frequently found under stress conditions [33], but since the expression of the more abundant Hxk2 protein did not change, the significance of this is unclear.

**Table 1:** **Proteins showing at least 1.5 fold overexpression with 95% significance in *JBA-mut *compared to *JBA-wt***

**Gene**	**Corresponding protein**	**Fold change**	**Mitochondrial location**
PIM1	Lon protease homolog	2.86	**X**
MAM33	Mitochondrial acidic protein MAM33	2.72	**X**
CYT1	Cytochrome c1, heme protein	2.09	**X**
GLO1	Lactoylglutathione lyase	2.01	
HSP42	Heat shock protein 42	1.97 *	
AIM2	Protein AIM2	1.94	**X**
FUN30	Uncharacterized ATP-dependent helicase FUN30	1.88 *	**X**
HOR2/GPP2	(DL)-glycerol-3-phosphatase 2	1.81 *	
MCR1	NADH-cytochrome b5 reductase 2	1.80 *	**X**
GLK1	Glucokinase-1	1.74 *	
MRPL38	54S ribosomal protein L38	1.70 *	**X**
QCR6	Cytochrome b-c1 complex subunit 6	1.67	**X**
EDE1	EH domain-containing and endocytosis protein 1	1.65	
MSS116	ATP-dependent RNA helicase MSS116	1.65 *	**X**
YPL088W	Putative aryl-alcohol dehydrogenase YPL088W	1.64 *	
ATP4	ATP synthase subunit 4	1.62	**X**
ATP17	ATP synthase subunit f	1.62 *	**X**
PEP4	Saccharopepsin	1.62	
LSP1	Sphingolipid long chain base-responsive protein LSP1	1.62	
QCR2	Cytochrome b-c1 complex subunit 2	1.61	**X**
COX4	Cytochrome c oxidase subunit 4	1.59	**X**
ZWF1	Glucose-6-phosphate 1-dehydrogenase	1.59	
ECM33	Cell wall protein ECM33	1.58	
GVP36	Protein GVP36	1.57	
CCP1	Cytochrome c peroxidase	1.57	**X**
CAR2	Ornithine aminotransferase	1.57 *	
AAC2	ADP, ATP carrier protein 2	1.56	**X**
CYC1	Cytochrome c iso-1	1.56 *	**X**
ATP1	ATP synthase subunit alpha	1.55 *	**X**
ATP2	ATP synthase subunit beta	1.54 *	**X**
CPR3	Peptidyl-prolyl cis-trans isomerase C	1.54	**X**
KGD1	2-oxoglutarate dehydrogenase	1.54	**X**
QCR7	Cytochrome b-c1 complex subunit 7	1.53	**X**
MRP8	Uncharacterized protein MRP8	1.51 *	

14 proteins remain upregulated also with 99% significant t-test (marked with asterisks).

**Table 2:** **Proteins showing at least 1.5 fold down-regulation with 95% significance in *JBA-mut *compared to *JBA-wt***

**Gene**	**Corresponding protein**	**Fold change**
TY2A-GR1	Transposon Ty2-GR1 Gag polyprotein	0.24
RPL7B	60S ribosomal protein L7-B	0.39 *
MET6	5-methyltetrahydropteroyltriglutamate--homocysteine methyltransferase	0.50 *
HIS1	ATP phosphoribosyltransferase	0.51
HRI1	Protein HRI1	0.56
TDH2	Glyceraldehyde-3-phosphate dehydrogenase 2	0.57 *
SHM2	Serine hydroxymethyltransferase, cytosolic	0.59
RTN1	Reticulon-like protein 1	0.60
PDC5	Pyruvate decarboxylase isozyme 2	0.62
STE20	Serine/threonine-protein kinase STE20	0.65
FSH1	Family of serine hydrolases 1	0.66
NOP16	Nucleolar protein 16	0.66 *

4 proteins remain downregulated also with 99% significant t-test (marked with asterisks).

In *E. coli* the chaperone GroESL has been implicated as a factor in butanol tolerance [37]. We do not see a strong upregulation of the yeast homolog of GroEL (Hsp60), but the most upregulated protein in our study is the Lon protease homolog Pim1, which is involved in degradation of misfolded protein in the mitochondrial matrix [38]. The cytoplasmic heat shock protein Hsp42 was also found as a highly significant, 2-fold induced protein in the *JBA-mut* strain. Hsp42 has a role in sorting of peripheral protein aggregates in *S. cerevisiae* under non-heat shock conditions causing protein folding defects, thereby aiding in maintaining protein homeostasis [39]. We have not looked into the formation of protein aggregates in our strain, but from analogy to the situation in *E. coli* we find it likely that butanol stress would lead to problems of protein folding and hence to aggregate formation, thereby providing a plausible explanation for the upregulation of Hsp42 and Pim1.

There were also 12 proteins showing a significant (95% confidence level by Student’s t-test) and ≥1.5 fold reduced abundance in the *JBA-mut* strain (Table 2). The protein showing the largest change was a transposon Ty2 Gag polyprotein, which could indicate that a transposon of this type has moved to a new location. However, although there are usually many transposons in the yeast genome [40], this was the only transposon associated protein detected in our samples, and the biological relevance of this finding is therefore hard to judge. We are planning to sequence the genomes of the *JBA-mut* and *JBA-wt* strains which might shed some light on this issue. The second most repressed protein was Rpl7B, a component of the large subunit of the ribosome. This was the only ribosomal protein showing a significant change in expression of >1.5 fold in any direction. Rpl7B is highly similar to Rpl7A which shows no change in expression. The significance of this differential expression is unclear, even though it has previously been suggested that the two isoforms may have somewhat different roles in the cell [41]. The repressed proteins Met6, His1 and Shm2 are all involved in amino acid biosynthesis indicating that there could be specific changes of fluxes over these reactions. However, the vast majority of amino acid biosynthetic enzymes do not display a significantly changed abundance in the *JBA-mut*. There are also two minor isoforms of proteins in the glycolysis and ethanol-synthetic pathway showing a lowered abundance in the *JBA-mut* strain: glyceraldehyde-3-phosphate dehydrogenase (Tdh2) and pyruvate decarboxylase (Pdc5). However, the corresponding dominant isoforms of these enzymes (Tdh3 and Pdc1) show no significant change in expression. Overall, the mechanistic significance of the proteins showing reduced abundance is thus unclear. Changing the t-test significance to ≥99% results in 14 proteins up-regulated (8 of which are mitochondrial) and 4 proteins down-regulated at least 1.5 fold (Table 1 and Table 2).

In summary, the main hypothesis emerging from the proteomics analysis is that a somewhat higher mitochondrial activity, perhaps together with a higher glycerol biosynthesis and a selection of stress response proteins (e.g. Hsp42 and Glo1) is the main factor underlying the butanol tolerant phenotype. This is in good agreement with previously published data on ethanol tolerance determinants which have also underscored the role of mitochondria [35,42,43]. Furthermore, protein degradation and retrograde signalling have recently been suggested as key factors in mediating butanol tolerance in yeast [44]. We find no evidence for increased proteolysis in our data, but it is possible that the increase we observe in mitochondrial proteins could be mechanistically linked to retrograde signalling as proposed by Gonzalez-Ramos et al. [44].

### Improvement of butanol tolerance by over expression of selected targets proposed from proteomics data

**Figure 2:**
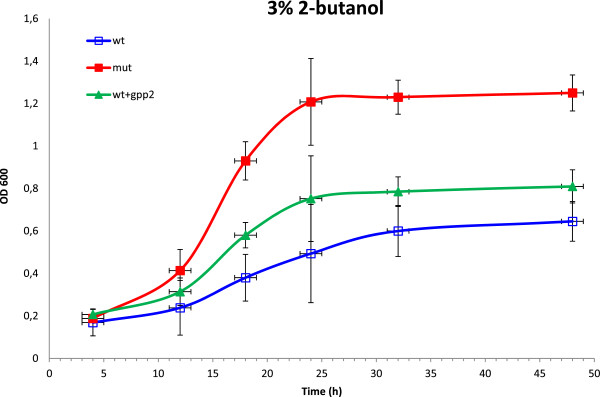
**Growth of *JBA-wt *(blue open squares), *JBA-wt + TDH3promotor-GPP2 *(green closed triangles) and *JBA-mut *(red closed squares) in YPD media supplemented with 3% (v/v) 2-butanol, respectively.** Three independent sets of cultivations were performed for each strain and error bars indicate the standard deviations.

To test the hypothesis that the upregulated proteins were at least in part responsible for the increased butanol tolerance of the mutant strain (*JBA-mut*), we decided to investigate into whether individual over-expression of the three most strongly regulated, non-mitochondrial, proteins (Gpp2, Glo1 and Hsp42; see Table 1) would lead to a tolerant phenotype. Overexpression was achieved by genomic integration of a construct in which the gene expression was placed under the control of a strong *TDH3* promotor (see Methods-section). The results showed that only the overexpression of *GPP2* mediated an increase in butanol tolerance at a 2-butanol concentration of 3% (v/v) although, as expected, not to the same level as the mutant (Figure 2). Overexpression of *GLO1* and *HSP42* did not improve the butanol tolerance of the wild-type strain (data not shown). We also tried overexpression of HAP4 which has been shown to induce an increase in respiration [45]. For unknown reasons we were unable to overexpress HAP4 in the *JBA-wt* strain, but the same construct when introduced into a wild type CEN_PK strain did not provide a butanol tolerant phenotype. This could indicate that either (i) the increase in mitochondrial activity proposed by the proteomics data is dependent on synergy with other changes in gene expression, presumably not regulated via Hap4 and furthermore not present in the haploid CEN_PK strain, or (ii) that the change in mitochondrial activity could be a consequence of the changes in gene/protein expression mediating the butanol tolerance. The synergy hypothesis would explain why most of the tested overexpressions did not lead to a tolerance phenotype, but it would also suggest studying overexpression of combinations of two or more proteins simultaneously which would rapidly turn into a daunting task given the number of candidate genes. However, since increased Gpp2 expression seems to be a contributing factor to butanol tolerance, it will be interesting in future studies to clarify the role of glycerol metabolism and its connection to mitochondrial processes (and other proteins found in this study) in mediating butanol tolerance.

Thus, since increased Gpp2 expression seems to be a contributing factor to butanol tolerance, it will be interesting in future studies to clarify the role of glycerol metabolism and its connection to mitochondrial processes in mediating butanol tolerance.

### Lipid analysis

**Figure 3:**
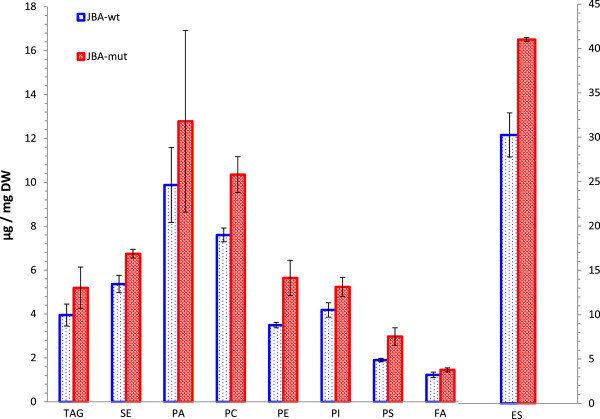
**Lipid and fatty acid composition of *JBA-wt *(blue open bars) and *JBA-mut *(red filled bars) during growth in YPD with 1.2% (v/v) 2-butanol.** Two independent sets of cultivations were performed for each strain and error bars indicate the min/max values. (TAG - triacylglycerol, SE - steryl ester, PA - Phosphatidic acid, PC - Phosphatidylcholine, PE - phosphatidyletanolamine, PI - phosphatidylinositol, PS - phosphatidylserine, FA - free fatty acid, ES - ergosterol).

Several studies have shown the connection of alcohol-stress and membrane lipid composition of cells [22,25,46-48]. We therefore decided to investigate whether the mutant phenotype was at least in part also dependent on changes in the abundance of one or several different classes of lipids. Following the procedure described by Khoomrung *et al.*[49] we could extract the lipids and fatty acids by using a single extraction. Similar to the proteomics samples the cells were grown in YPD with 1.2% v/v 2-butanol and harvested at OD_600_ ≈ 1. There was a general trend showing a somewhat increased content of lipids in the *JBA mut* strain (Figure 3), however, these changes were not found significant by a Student t-test. The largest increase was obtained for phosphatidylethanolamine (PE) and phosphatidylserine (PS) where an increase of about 60% was observed for the mutant compared to the wild-type.

However, we have not looked at the composition of the constituent fatty acids for each class of lipid. Thus, although the abundance of the various classes of lipids does not change significantly, the properties of the fatty acid composition in some or all classes might do so. These analyses would be interesting to perform as a follow up study. However, at present we find little evidence for involvement of lipids in mediating the tolerant phenotype of the mutant, both from the lack of changes in enzymes in lipid biosynthesis and from direct lipid measurements.

### Physiological growth characteristics

**Figure 4:**
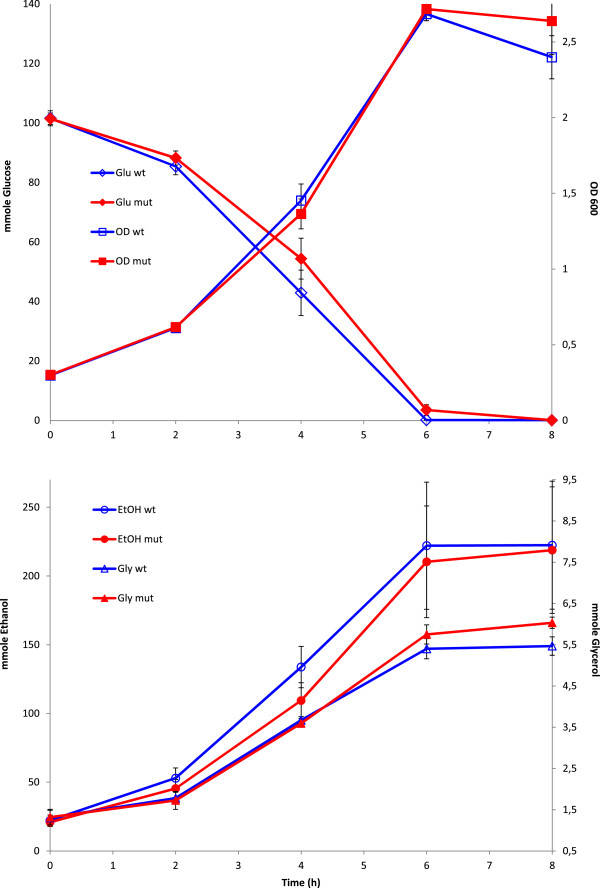
**Growth characteristics of *JBA-wt *(blue open symbols) and *JBA-mut *(red closed symbols).** Cells were grown in YPD, in the presence of 1.2% 2-butanol (v/v). Measured variables were optical density (squares), glucose (diamonds), ethanol (circles) and glycerol (triangles). Two independent sets of cultivations were performed for each strain and error bars indicate the min/max values.

Some differences in growth characteristics were discovered when cultivating *JBA-wt* and *JBA-mut* in bioreactors, in the presence of 1.2% (v/v) 2-butanol. As described before, the growth rate at this butanol concentration was more or less identical between the two strains (Figure 4). The growth yield and glucose consumption rates are also quite similar though there was a slightly reduced sugar consumption rate and somewhat higher final OD of the mutant. There was also a small difference in fermentation rates as the mutant displayed reduced ethanol production rate compared to the wild-type (Figure 4). This is consistent with the proteomics data which indicated a higher mitochondrial activity of the mutant. Another observation that could be related to changes in protein levels was the increase in glycerol production in the mutant (Figure 4) since glycerol 3-phosphatase 2 (Gpp2) was one of the proteins showing the highest and most significant (≥99% significance) up-regulation. In many cases an increase in ethanol production rate is also accompanied by an increase in glycerol formation [50] but in this case the evolved tolerant mutant may have induced an inherent higher glycerol production rate as a general stress response.

It should be also mentioned that the *JBA-mut* did not display a butanol tolerant phenotype during growth under anaerobic conditions (data not shown). An increased glycerol production rate is well known to occur under anaerobic conditions [51], but the restricted ATP synthesizing capacity under this condition most likely prevents the ability to tolerate high butanol concentrations.

## Conclusion

The study showed that the wild-type and the mutant differed in a number of aspects. Based on the proteomics data, which are consistent with the physiological characterization, higher mitochondrial activity of the *JBA-mut,* together with an increased expression of the glycerol 3-phosphatase isoform Gpp2 and increased expression of some of the stress metabolic enzymes (e.g. Glo1 and Hsp42), seem to be the main characteristics providing the *JBA-mut* with the observed increase in butanol tolerance. The mechanistic explanation for the tolerance phenotype is likely to depend on synergistic effects of several regulatory changes, which we at present do not fully understand, but which are presently being studied in our group.

The fact that overexpression of *GPP2* in *JBA-wt* helped it to adapt to butanol stress further corroborates the role of Gpp2.

The tolerance mechanisms proposed in the present work would lead to a somewhat higher flow of carbon to glycerol and likely also to respiration, thereby to some extent reducing the yield of butanol. However, in the case of most biofuel production processes, the end product toxicity has been identified as a major problem [14]. Therefore, in an industrial process to produce 2-butanol, this could be acceptable, since even a rather modest increase in tolerance to butanol would have a significant value, due to the rather high toxicity of 2-butanol.

## Methods

### Strain

An industrial strain of *S. cerevisiae*, obtained from the Baker’s Yeast Company in Rotebro, Sweden, was used as the starting strain (*JBA-wt*).

### Cultivation

The cells were grown in YPD medium (20 g/l glucose, 20 g/l peptone and 10 g/l yeast extract). Through 30 sequential batch cultures in 50 ml falcon tubes the mutated strain was obtained. Butanol concentration was gradually increased along the transfers and prior to each transfer, cells were recovered in fresh YPD medium for 30 minutes. Each time 0.5 ml of culture (approximately 1.5 x 10^7^ cells) was transferred.

Later on the wild type (*JBA-wt*) and the mutant (*JBA-mut*) strains were cultivated in bioreactors (Dasgip, Jülich, Germany) (in the presence of 10 g/l 2-butanol) for characterization. The medium was stirred at 400 rpm and no air was purged to avoid stripping of butanol.

### Strain construction and transformation

The open reading frames from the genes of interest (i.e. *GPP2, GLO1* and *HSP42*) were integrated into the genome of *JBA-wt* using an integrative construct (provided as Additional file 1) at a locus 1000 bp downstream of the DAK2 gene stop-codon with the Kanamycin resistance marker (KanMX4) used to isolate correct colonies. Standard lithium acetate protocol was used for transformation.

### MS sample preparation

80 μl of cell pellets were mixed with 70 μl 100 mM Triethylammoniumbicarbonate (TEAB) solution to keep the pH stable at about 8. 50 μl glassbeads was added and the sample was mixed vigorously by Fast Prep (MP Biomedicals Solon, OH, USA) to break the cells (20 seconds at speed 6 for 4 times, kept 30 seconds on ice between every run). 30 μl 10% SDS was added and the samples were heated at 60°C for 5 minutes. The samples were centrifuged at high speed for 2 minutes and the supernatant was kept.Total protein concentration was determined using Pierce 660 nm Protein Assay (Thermo Scientific). Three samples in each group (mutant and wildtype) containing 100 μg protein in each sample were incubated with TCEP (tris(2-carboxyethyl)phosphine) and then transferred to 3 K mw cutoff filters (PALL) and diluted with 8 M urea in 0.1 M TEAB (triethyl ammonium bicarbonate) for filter aided sample preparation (FASP) [52].

Samples were alkylated with MMTS (methyl methanethiosulfonate) and digested with trypsin in 0.5 M TEAB ratio 1:25 over night in 37 C. The peptides were eluted at 12000 rpm for 10 min and filters were rinsed with 20% ACN (acetonitrile) at 12000 rpm for 5 min.

### Label with TMT reagents

TMT reagents 126, 128 and 130 (wildtype samples) and 127, 129 and 131 (mutant samples) was dissolved in ACN and added to the respectively sample according to manufacturer’s protocol (Thermo Fisher Scientific). After labelling and quenching of the reagents, the samples were combined and concentrated. TMT-labelled peptides were separated with Strong Cation Exchange Chromatography (SCX). The concentrated peptides were acidified by 10% formic acid and diluted with SCX solvent A (25 mM ammonium formate, pH 2.8, 20% ACN and injected onto a PolySULFOETHYL A SCX column (2.1 mm i.d. × 10 cm length, 5 μm particle size, 300 Å pore size). SCX chromatography and fractionation was carried out on an ÄKTA purifier system (GE healthcare) at 0.25 mL/min flow rate using the following gradient: 0% B (500 mM ammonium formate, pH 2.8, 20% ACN) for 5 min; 0-20% B for 20 min; 20-40% B for 10 min and 40-100% B for 10 min. UV absorbance at 254 and 280 nm was monitored while fractions were collected at 0.5 mL intervals and dried down in a SpeedVac. The 18 peptide containing fractions were desalted on PepClean C18 spin columns according to manufacturer’s instructions (Thermo Fisher Scientific).

### LC-MS/MS analysis on LTQ-OrbitrapXL

The desalted and dried fractions were reconstituted into 0.1% formic acid and analyzed on a LTQ-OrbitrapXL (Thermo Fisher Scientific) interfaced with an in-house constructed nano-LC column. Two-micro liter sample injections were made with an HTC-PAL autosampler (CTC Analytics AG) connected to an Agilent 1200 binary pump (Agilent Technologies). The peptides were trapped on a pre-column (40 x 0.075 mm i.d.) and separated on a reversed phase column, 200 x 0.075 μm. Both columns are packed in-house with 3 μm Reprosil-Pur C18-AQ particles. The flow through the analytical column was reduced by a split to approximately 200 nl/min and the gradient was as followed; 0–6 min 0.1% formic acid, 6–106 min 5-37% ACN, 0.1% formic acid, up to 80% ACN during 3 min and hold at 80% for 5 min.

LTQ-OrbitrapXL settings were: spray voltage 1.4 kV, 1 microscan for MS1 scans at 60 000 resolutions (m/z 400), full MS mass range m/z 400–2000. The LTQ-OrbitrapXL was operated in a data-dependent mode with one MS1 FTMS scan of precursor ions followed by CID (collision induced dissociation) and HCD (high energy collision dissociation), MS2 scans of the three most abundant doubly, triply and quadruply protonated ions in each FTMS scan. The settings for the MS2 were as follows: 1 microscans for HCD-MS2 at 7500 resolution (at m/z 400), mass range m/z 100–2000 with a collision energy of 50%, 1 microscans for CID-MS2 with a collision energy of 30%. Dynamic exclusion of a precursor selected for MS2 was used for 30s after one repeat, enabling most of the co-eluting precursors to be selected for MS2.

### Database search and TMT quantification

MS raw data files from all SCX fractions for the TMT set were merged for relative quantification and identification using Proteome Discoverer version 1.3 (Thermo Fisher Scientific). Database search was performed by Mascot search engine using the following critera: *Saccharomyces cerevisiae* in Swissprot protein database (version may 2012), MS peptide tolerance as 10 ppm, MS/MS tolerance as 0.5 Da, trypsin digestion allowing 1 missed cleavages with variable modifications; methionine oxidation, cysteine methylthiol, and fixed modifications; N-terminal TMT-label, lysine TMT-label. The detected protein threshold in the software was set to 99% confidence and identified proteins were grouped by sharing the same sequences to minimize redundancy.

For TMT quantification, the ratios of TMT reporter ion intensities in MS/MS spectra (m/z 126.12, 127.13, 128.13, 129.14, 130.14, 131.14) from raw data sets were used to calculate fold changes between samples. The average of all three reporters for the wild-type samples was used as the denominator. Only peptides unique for a given protein were considered for relative quantitation, excluding those common to other isoforms or proteins of the same family. The resulting ratios were then exported into Excel (Microsoft) for further data interpretation.

### Extraction and analysis of lipids and fatty acids

Lipids (including steryl ester (SE), ergosterol (ES), triacylglycerol (TAG), phosphatidic acid (PA), phosphatidylethanolamine (PE), phosphatidylcholine (PC), phosphatidylserine (PS) and phosphatidylinositol (PI)) and fatty acids were extracted based on a microwave-assisted method [49]. About 10 mg of freeze-dried cells together with internal standard were mixed with chloroform-methanol. N_2_ gas was purged and samples were heated up in microwave reaction vessel. Samples were mixed with 0.73% w/v NaCl solution and centrifuged afterwards. Samples were concentrated under vacuum drying and then resuspended in chloroform-methanol. Detailed procedure can be found in [49].

Lipid analysis was done according to [49] by HPLC (Dionex; ultimate 3000 HPLC system, Germany) and a CAD detector (Corona; ESA, Chelmsford, MA, USA) streamed with N_2_ at 35 psi. 2 μl of sample was passed through a Luna 5 mm HILIC 100 Å (250 x 4.6 mm) LC Column (Phenomenex). The flow rate of 0.8 mL/min was applied and temperature was kept at 35°C. Three solution of (1) hexane-acetic acid (99:1, v/v); (2) acetone-isopropanol-acetic acid (29:70:1, v/v) and (3) water-acetone-isopropanol-acetic acid (9:20:70:1, v/v) were used as mobile phase and pH was adjusted to 5 by addition of triethylamine (0.08%, v/v).

The sample was run for 45.9 minutes and the starting elution solution was 100% solvent (1) at t = 0 min. Solvent (2) and solvent (3) were added gradually and fraction of solvent (1) was altered accordingly. The gradient of solvent (2) was as follow: 1% (t = 5 min), 2% (t = 6 min), 3% (t = 14 min), 5% (t = 19-36 min), 20% (t = 38 min), 2% (t = 40 min) and 0% (t = 42 min). The gradient of solvent (3) reached the following: 0.5% (t = 14 min), 35% (t = 19 min), 44% (t = 36 min) and 0% (t = 38 min). Lipids were quantified using external calibration curves. Lipid standards with known concentrations (ranging from 10 to 1000 μg/mL) were used.

### Analysis of extracellular metabolites

Metabolites such as glucose, ethanol, glycerol and acetate were analyzed by HPLC (Ultimate 3000, Dionex, Sunnyvale, US) through an Aminex® HPX-87H column (300 x 7.8 mm) (Bio-Rad, Hercules, CA, USA). 5 mM H_2_SO_4_ was used as eluent at 0.6 ml/min and the running temperature was 45°C.

## Abbreviations

ACN: Acetonitrile; CAD: Charged aerosol detector; CID: Collision induced dissociation; DAK2: Dihydroxy acetone kinase; ES: Ergosterol; FASP: Filter aided sample preparation; FTMS: Fourier transform mass spectrometry; HAP4: Heme activator protein; HPLC: High-performance liquid chromatography; HCD: High energy collision dissociation; HXK2: Hexokinase; JBA-mut: Mutated strain; JBA-wt: Wild type strain; LC: Liquid chromatography; MMTS: Methyl methanethiosulfonate; MS: Mass spectrometry; PA: Phosphatidic acid; PC: Phosphatidylcholine; PE: Phosphatidylethanolamine; PI: Phosphatidylinositol; PS: Phosphatidylserine; SCX: Strong cation exchange chromatography; SE: Steryl ester; TCEP: Tris(2-carboxyethyl)phosphine; TDH3: Triosephosphate dehydrogenase; TEAB: Triethylammoniumbicarbonate; TMT: Tandem mass tag; TAG: Triacylglycerol.

## Competing interests

The authors declare that they have no competing interests.

## Authors’ contributions

PG performed all the experiments and took part in planning, writing and interpretation of results. JN and CL took part in planning, writing and interpretation of results. All authors read and approved the final manuscript.

## Supplementary Material

Additional file 1 **Sequence of the integrative constructs used to overexpress Gpp2, Glo1 and Hsp42 in *JBA-wt.*** Sequences in orange are the regions of homology to the *DAK2* downstream region. The relevant open reading frame to be overexpressed is shown in blue, with start and stop codons highlighted yellow. Click here for file
