# Supplementary material for: Physiological adaptations of Saccharomyces cerevisiae evolved for improved butanol tolerance
Source: Biotechnol Biofuels. 2013 Jul 15;6:101. doi: 10.1186/1754-6834-6-101 (PMC3729582; doi:10.1186/1754-6834-6-101)
Supplement: Additional file 1 — Sequence of the integrative constructs used to overexpress Gpp2, Glo1 and Hsp42 in JBA-wt. Sequences in orange are the regions of homology to the DAK2 downstream region. The relevant open reading frame to be overexpressed is shown in blue, with start and stop codons highlighted yellow. [file 1754-6834-6-101-S1.pdf]

Supplementary information for paper by Ghiaci *et al*.

Sequences of the PCR-fragments used to integrate overexpression constructs of *GPP2, GLO1* and *HSP42* at a locus 1000 bp downstream of the *DAK2* stop-codon. All three constructs have a 574 bp *TDH3* promotor and a 229 bp 3'UTR/terminator region from *ADH1*.

Sequence of the *GPP2* overexpression construct:

TTCATGCATCTAAGAAATCAACCTATATCAACAGATTTCAATAATTACTCTAAACTT
ATGCTGTAACTTAGAAAGTAACCAGCCTGTGTTGACTGATTGAGTTGCGTATTAACT
GCGCCTAGTCATTTCAACACTTATAATTTGCTTCAGCTTAAGTGTGGTTCATCTTTT
TTTTTCTGGAAACTTTGCATGCCCTCAAAgtcgacCTGCTGTAACCCGTACATGCCC
AAAATAGGGGGCGGGTTACACAGAATATATAACATCGTAGGTGTCTGGGTGAACAGT
TTATTCCTGGCATCCACTAAATATAATGGAGCCCGCTTTTTAAGCTGGCATCCAGAA
AAAAAAAGAATCCCAGCACCAAAATATTGTTTTCTTCACCAACCATCAGTTCATAGG
TCCATTCTCTTAGCGCAACTACAGAGAACAGGGGCACAAACAGGCAAAAAACGGGCA
CAACCTCAATGGAGTGATGCAACCTGCCTGGAGTAAATGATGACACAAGGCAATTGA
CCCACGCATGTATCTATCTCATTTTCTTACACCTTCTATTACCTTCTGCTCTCTCTG
ATTTGGAAAAAGCTGAAAAAAAAGGTTGAAACCAGTTCCCTGAAATTATTCCCCTAC
TTGACTAATAAGTATATAAAGACGGTAGGTATTGATTGTAATTCTGTAAATCTATTT
CTTAAACTTCTTAAATTCTACTTTTATAGTTAGTCTTTTTTTTAGTTTTAAAACACC
AAGAACTTAGTTTCGAATAAACACACATAAACAAACAAAggatccATGGGATTGACT
ACTAAACCTCTATCTTTGAAAGTTAACGCCGCTTTGTTCGACGTCGACGGTACCATT
ATCATCTCTCAACCAGCCATTGCTGCATTCTGGAGGGATTTCGGTAAGGACAAACCT
TATTTCGATGCTGAACACGTTATCCAAGTCTCGCATGGTTGGAGAACGTTTGATGCC
ATTGCTAAGTTCGCTCCAGACTTTGCCAATGAAGAGTATGTTAACAAATTAGAAGCT
GAAATTCCGGTCAAGTACGGTGAAAAATCCATTGAAGTCCCAGGTGCAGTTAAGCTG
TGCAACGCTTTGAACGCTCTACCAAAAGAGAAATGGGCTGTGGCAACTTCCGGTACC
CGTGATATGGCACAAAAATGGTTCGAGCATCTGGGAATCAGGAGACCAAAGTACTTC
ATTACCGCTAATGATGTCAAACAGGGTAAGCCTCATCCAGAACCATATCTGAAGGGC
AGGAATGGCTTAGGATATCCGATCAATGAGCAAGACCCTTCCAAATCTAAGGTAGTA
GTATTTGAAGACGCTCCAGCAGGTATTGCCGCCGGAAAAGCCGCCGGTTGTAAGATC
ATTGGTATTGCCACTACTTTCGACTTGGACTTCCTAAAGGAAAAAGGCTGTGACATC
ATTGTCAAAAACCACGAATCCATCAGAGTTGGCGGCTACAATGCCGAAACAGACGAA
GTTGAATTCATTTTTGACGACTACTTATATGCTAAGGACGATCTGTTGAAATGGTAA
ggcgcgccACTTCTAAATAAGCGAATTTCTTATGATTTATGATTTTTATTATTAAAT
AAGTTATAAAAAAAATAAGTGTATACAAATTTTAAAGTGACTCTTAGGTTTTAAAAC
GAAAATTCTTATTCTTGAGTAACTCTTTCCTGTAGGTCAGGTTGCTTTCTCAGGTAT
AGTATGAGGTCGCTCTTATTGACCACACCTCTACCGGCAGATCCGCTAGGGATAACA
GGGTAATATgagctcataacttcgtataatgtatgctatacgaagttatgcggccgc
taggtctagagatctgtttagcttgcctcgtccccgccgggtcacccggccagcgac
atggaggcccagaataccctccttgacagtcttgacgtgcgcagctcaggggcatga
tgtgactgtcgcccgtacatttagcccatacatccccatgtataatcatttgcatcc
atacattttgatggccgcacggcgcgaagcaaaaattacggctcctcgctgcagacc
tgcgagcagggaaacgctcccctcacagacgcgttgaattgtccccacgccgcgccc
ctgtagagaaatataaaaggttaggatttgccactgaggttcttctttcatatactt
ccttttaaaatcttgctaggatacagttctcacatcacatccgaacataaacaacc**a**
tgggtaaggaaaagactcacgtttcgaggccgcgattaaattccaacatggatgctg
atttatatgggtataaatgggctcgcgataatgtcgggcaatcaggtgcgacaatct
atcgattgtatgggaagcccgatgcgccagagttgtttctgaaacatggcaaaggta
gcgttgccaatgatgttacagatgagatggtcagactaaactggctgacggaattta
tgcctcttccgaccatcaagcattttatccgtactcctgatgatgcatggttactca
ccactgcgatccccggcaaaacagcattccaggtattagaagaatatcctgattcag
gtgaaaatattgttgatgcgctggcagtgttcctgcgccggttgcattcgattcctg
tttgtaattgtccttttaacagcgatcgcgtatttcgtctcgctcaggcgcaatcac

gaatgaataacggtttggttgatgcgagtgattttgatgacgagcgtaatggctggc
ctgttgaacaagtctggaaagaaatgcataagcttttgccattctcaccggattcag
tcgtcactcatggtgatttctcacttgataaccttatttttgacgaggggaaattaa
taggttgtattgatgttggacgagtcggaatcgcagaccgataccaggatcttgcca
tcctatggaactgcctcggtgagttttctccttcattacagaaacggctttttcaaa
aatatggtattgataatcctgatatgaataaattgcagtttcatttgatgctcgatg
agtttttc**taa**tcagtactgacaataaaaagattcttgttttcaagaacttgtcatt
tgtatagtttttttatattgtagttgttctattttaatcaaatgttagcgtgattta
tatttttttcgcctcgacatcatctgcccagatgcgaagttaagtgcgcagaaagt
aatatcatgcgtcaatcgtatgtgaatgctggtcgctatactgctgtcgattcgata
ctaacgccgccatccagtgtcgaaaacgagctttcgagaacccttaatgcggccgca
taacttcgtataatgtatgctatacgaagttatGCATGAGTAGTTAGTTATCTTTTT
GACAATGATCTCTTTTGAAAATATCTACTGTAGATTTGCATGGACGCACGTCGCCCA
TACGCCAAACTTTGGCAATGATACTCGTTATTCGTAATATCAGTCCGTCAAGGTGCT
GTGATTTCTCTATTTTATATTGCCTATTATTTTTTCAAATGATTTGAGCCGTTTTAA
ATTGA

Sequence of the *GLO1* overexpression construct:

TTCATGCATCTAAGAAATCAACCTATATCAACAGATTTCAATAATTACTCTAAACTT
ATGCTGTAACTTAGAAAGTAACCAGCCTGTGTTGACTGATTGAGTTGCGTATTAACT
GCGCCTAGTCATTTCAACACTTATAATTTGCTTCAGCTTAAGTGTGGTTCATCTTTT
TTTTTCTGGAAACTTTGCATGCCCTCAAAgtcgacCTGCTGTAACCCGTACATGCCC
AAAATAGGGGGCGGGTTACACAGAATATATAACATCGTAGGTGTCTGGGTGAACAGT
TTATTCCTGGCATCCACTAAATATAATGGAGCCCGCTTTTTAAGCTGGCATCCAGAA
AAAAAAAGAATCCCAGCACCAAAATATTGTTTTCTTCACCAACCATCAGTTCATAGG
TCCATTCTCTTAGCGCAACTACAGAGAACAGGGGCACAAACAGGCAAAAAACGGGCA
CAACCTCAATGGAGTGATGCAACCTGCCTGGAGTAAATGATGACACAAGGCAATTGA
CCCACGCATGTATCTATCTCATTTTCTTACACCTTCTATTACCTTCTGCTCTCTCTG
ATTTGGAAAAAGCTGAAAAAAAAGGTTGAAACCAGTTCCCTGAAATTATTCCCCTAC
TTGACTAATAAGTATATAAAGACGGTAGGTATTGATTGTAATTCTGTAAATCTATTT
CTTAAACTTCTTAAATTCTACTTTTATAGTTAGTCTTTTTTTTAGTTTTAAAACACC
AAGAACTTAGTTTCGAATAAACACACATAAACAAACAAAggatctATGTCCACTGAT
AGTACACGCTATCCAATTCAGATTGAGAAAGCCTCGAATGATCCAACCCTTCTGCTT
AATCACACATGTTTAAGAGTCAAGGATCCAGCAAGGACCGTTAAGTTCTACACCGAA
CACTTCGGTATGAAGCTATTAAGCAGAAAGGATTTTGAAGAAGCAAAATTTAGCTTG
TACTTTTTAAGCTTTCCAAAAGACGACATACCCAAAAATAAGAATGGAGAGCCTGAT
GTTTTTAGCGCACACGGTGTCTTAGAACTAACTCACAATTGGGGTACTGAAAAAAAC
CCAGACTACAAGATCAACAACGGGAATGAGGAACCTCATCGTGGATTTGGGCACATC
TGTTTTTCTGTATCCGATATCAATAAAACCTGCGAAGAGCTAGAATCTCAGGGTGTC
AAATTCAAGAAGAGACTCTCTGAAGGAAGACAGAAGGACATTGCGTTTGCTTTAGGC
CCTGATGGATACTGGATTGAGTTGATCACATATTCTAGAGAGGGTCAGGAATACCCA
AAGGGCTCAGTAGGTAACAAGTTCAATCATACCATGATTCGTATTAAAAACCCAACC
CGGTCTTTAGAATTCTACCAGAATGTGTTGGGCATGAAATTATTAAGAACTAGTGAG
CACGAAAGTGCAAAATTTACGTTATACTTTCTTGGTTATGGCGTTCCAAAGACCGAC
AGCGTTTTTTCATGTGAAAGTGTGTTGGAGTTAACTCATAATTGGGGAACTGAGAAT
GATCCAAACTTCCACTATCATAACGGTAACTCAGAGCCCCAGGGTTATGGTCACATC
TGCATAAGTTGTGATGACGCTGGCGCCCTTTGTAAAGAAATTGAAGTGAAATACGGC
GATAAGATCCAATGGTCTCCTAAATTTAACCAAGGCAGAATGAAGAATATTGCCTTT
TTGAAGGATCCTGATGGTTATTCCATTGAAGTCGTTCCTCATGGTTTGATTGCCTAA
ggcgcgccACTTCTAAATAAGCGAATTTCTTATGATTTATGATTTTTATTATTAAAT
AAGTTATAAAAAAAATAAGTGTATACAAATTTTAAAGTGACTCTTAGGTTTTAAAAC
GAAAATTCTTATTCTTGAGTAACTCTTTCCTGTAGGTCAGGTTGCTTTCTCAGGTAT
AGTATGAGGTCGCTCTTATTGACCACACCTCTACCGGCAGATCCGCTAGGGATAACA
GGGTAATATgagctcataacttcgtataatgtatgctatacgaagttatgcggccgc
taggtctagagatctgtttagcttgcctcgtccccgccgggtcacccggccagcgac
atggaggcccagaataccctccttgacagtcttgacgtgcgcagctcaggggcatga

tgtgactgtcgcccgtacatttagcccatacatccccatgtataatcatttgcatcc
atacattttgatggccgcacggcgcgaagcaaaaattacggctcctcgctgcagacc
tgcgagcagggaaacgctcccctcacagacgcgttgaattgtccccacgccgcgccc
ctgtagagaaatataaaaggttaggatttgccactgaggttcttctttcatatactt
cctttaaaatcttgctaggatacagttctcacatcacatccgaacataaacaacc**a**
tgggtaaggaaaagactcacgtttcgaggccgcgattaaattccaacatggatgctg
atttatatgggtataaatgggctcgcgataatgtcgggcaatcaggtgcgacaatct
atcgattgtatgggaagcccgatgcgccagagttgtttctgaaacatggcaaaggta
gcgttgccaatgatgttacagatgagatggtcagactaaactggctgacggaattta
tgcctcttccgaccatcaagcattttatccgtactcctgatgatgcatggttactca
ccactgcgatccccggcaaaacagcattccaggtattagaagaatatcctgattcag
gtgaaaatattgttgatgcgctggcagtgttcctgcgccggttgcattcgattcctg
tttgtaattgtccttttaacagcgatcgcgtatttcgtctcgctcaggcgcaatcac
gaatgaataacggtttggttgatgcgagtgattttgatgacgagcgtaatggctggc
ctgttgaacaagtctggaaagaaatgcataagcttttgccattctcaccggattcag
tcgtcactcatggtgatttctcacttgataaccttatttttgacgaggggaaattaa
taggttgtattgatgttggacgagtcggaatcgcagaccgataccaggatcttgcca
tcctatggaactgcctcggtgagttttctccttcattacagaaacggctttttcaaa
aatatggtattgataatcctgatatgaataaattgcagtttcatttgatgctcgatg
agtttttc**taa**tcagtactgacaataaaaagattcttgttttcaagaacttgtcatt
tgtatagtttttttatattgtagttgttctattttaatcaaatgttagcgtgattta
tatttttttttcgcctcgacatcatctgcccagatgcgaagttaagtgcgcagaaagt
aatatcatgcgtcaatcgtatgtgaatgctggtcgctatactgctgtcgattcgata
ctaacgccgccatccagtgtcgaaaacgagctttcgagaacccttaatgcggccgca
taacttcgtataatgtatgctatacgaagttatGCATGAGTAGTTAGTTATCTTTTT
GACAATGATCTCTTTTGAAAATATCTACTGTAGATTTGCATGGACGCACGTCGCCCA
TACGCCAAACTTTGGCAATGATACTCGTTATTCGTAATATCAGTCCGTCAAGGTGCT
GTGATTTCTCTATTTTATATTGCCTATTATTTTTTCAAATGATTTGAGCCGTTTTAA
ATTGA

Sequence of the *HSP42* overexpression construct:

TTCATGCATCTAAGAAATCAACCTATATCAACAGATTTCAATAATTACTCTAAACTT
ATGCTGTAACTTAGAAAGTAACCAGCCTGTGTTGACTGATTGAGTTGCGTATTAACT
GCGCCTAGTCATTTCAACACTTATAATTTGCTTCAGCTTAAGTGTGGTTCATCTTTT
TTTTTCTGGAAACTTTGCATGCCCTCAAAgtcgacCTGCTGTAACCCGTACATGCCC
AAAATAGGGGGCGGGTTACACAGAATATATAACATCGTAGGTGTCTGGGTGAACAGT
TTATTCCTGGCATCCACTAAATATAATGGAGCCCGCTTTTTAAGCTGGCATCCAGAA
AAAAAAAGAATCCCAGCACCAAAATATTGTTTTCTTCACCAACCATCAGTTCATAGG
TCCATTCTCTTAGCGCAACTACAGAGAACAGGGGCACAAACAGGCAAAAAACGGGCA
CAACCTCAATGGAGTGATGCAACCTGCCTGGAGTAAATGATGACACAAGGCAATTGA
CCCACGCATGTATCTATCTCATTTTCTTACACCTTCTATTACCTTCTGCTCTCTCTG
ATTTGGAAAAAGCTGAAAAAAAAAGGTTGAAACCAGTTCCCTGAAATTATTCCCCTAC
TTGACTAATAAGTATATAAAGACGGTAGGTATTGATTGTAATTCTGTAAATCTATTT
CTTAAACTTCTTAAATTCTACTTTTATAGTTAGTCTTTTTTTTTAGTTTTAAAACACC
AAGAACTTAGTTTCGAATAAACACACATAAACAAACAAAggatccATGAGTTTTTAT
CAACCATCCCTATCTCTTTATGACGTTTTGAACGCATTATCCAACCAAACTGGCCAG
AGAGGGCAGCAAGGATATCCTCGCCAACCACAAAGGCCACAGAGATACCATCCCCAT
TATGGACAAGTGCACGTTGGCGGGCATCATCCTCGTCATCATCCATTGTATAGCAGA
TACAATGGTGTTCCTAATACCTATTACTACCAGTTCCCTGGACAAGCCTATTACTAT
AGTCCTGAATACGGTTATGATGACGAGGATGGTGAAGAAGAGGACCAAGACGAAGAT
ATGGTGGGTGACAGCGGCACTACAAGACAGGAAGATGGTGGCGAGGACAGCAACTCG
AGAAGATATCCATCATATTACCATTGTAATACTGCCAGGAATAATAGGACCAACCAA
CAGGCAAACAGTTTAAACGACTTATTAACCGCGTTAATAGGTGTTCCACCATATGAA
GGCACTGAACCAGAAATTGAAGCAAATACCGAACAGGAGGGCGAAAAGGGAGAAGAA
AAGGATAAGAAGGATAAGTCTGAAGCACCCAAAGAGGAAGCTGGCGAAACCAACAAA

GAAAAACCTTTGAATCAGCTGGAGGAATCGTCGAGACCACCATTAGCCAAAAAATCT
TCATCGTTCGCTCACCTACAAGCGCCTTCCCCAATACCTGACCCGTTACAAGTATCC
AAGCCTGAAACGAGAATGGACTTACCATTTTCACCAGAAGTGAATGTCTATGATACC
GAGGACACTTACGTAGTTGTTCTTGCGTTACCAGGTGCTAACTCTAGGGCTTTCCAC
ATTGATTACCATCCATCTTCTCATGAGATGCTCATCAAGGGTAAGATCGAAGACAGA
GTGGGCATTGATGAAAAATTCTTGAAGATCACGGAACTAAAATATGGTGCGTTTGAG
AGAACCGTTAAATTCCCCGTGCTGCCACGCATTAAGGACGAAGAAATTAAAGCTACT
TACAACAACGGTCTACTACAAATTAAGGTGCCTAAAATTGTCAATGACACTGAAAAG
CCGAAGCCAAAAAAGAGGATCGCCATTGAGGAAATACCCGACGAAGAATTGGAGTTT
GAAGAAAATCCCAACCCTACGGTAGAAAATTAAggcgcgccACTTCTAAATAAGCGA
ATTTCTTATGATTTATGATTTTTATTATTAAATAAGTTATAAAAAAAATAAGTGTAT
ACAAATTTTAAAGTGACTCTTAGGTTTTAAAACGAAAATTCTTATTCTTGAGTAACT
CTTTCCTGTAGGTCAGGTTGCTTTCTCAGGTATAGTATGAGGTCGCTCTTATTGACC
ACACCTCTACCGGCAGATCCGCTAGGGATAACAGGGTAATATgagctcataacttcg
tataatgtatgctatacgaagttatgcggccgctaggtctagagatctgtttagctt
gcctcgtccccgccgggtcacccggccagcgacatggaggcccagaataccctcctt
gacagtcttgacgtgcgcagctcaggggcatgatgtgactgtcgcccgtacatttag
cccatacatccccatgtataatcatttgcatccatacattttgatggccgcacggcg
cgaagcaaaaattacggctcctcgctgcagacctgcgagcagggaaacgctcccctc
acagacgcgttgaattgtccccacgccgcgcccctgtagagaaatataaaaggttag
gatttgccactgaggttcttctttcatatacttccttttaaaatcttgctaggatac
agttctcacatcacatccgaacataaacaacc**atg**ggtaaggaaaagactcacgttt
cgaggccgcgattaaattccaacatggatgctgatttatatgggtataaatgggctc
gcgataatgtcgggcaatcaggtgcgacaatctatcgattgtatgggaagcccgatg
cgccagagttgtttctgaaacatggcaaaggtagcgttgccaatgatgttacagatg
agatggtcagactaaactggctgacggaatttatgcctcttccgaccatcaagcatt
ttatccgtactcctgatgatgcatggttactcaccactgcgatccccggcaaaacag
cattccaggtattagaagaatatcctgattcaggtgaaaatattgttgatgcgctgg
cagtgttcctgcgccggttgcattcgattcctgtttgtaattgtccttttaacagcg
atcgcgtatttcgtctcgctcaggcgcaatcacgaatgaataacggtttggttgatg
cgagtgattttgatgacgagcgtaatggctggcctgttgaacaagtctggaaagaaa
tgcataagcttttgccattctcaccggattcagtcgtcactcatggtgatttctcac
ttgataaccttatttttgacgaggggaaattaataggttgtattgatgttggacgag
tcggaatcgcagaccgataccaggatcttgccatcctatggaactgcctcggtgagt
tttctccttcattacagaaacggctttttcaaaaatatggtattgataatcctgata
tgaataaattgcagtttcatttgatgctcgatgagtttttc**taa**tcagtactgacaa
taaaaagattcttgttttcaagaacttgtcatttgtatagtttttttatattgtagt
tgttctattttaatcaaatgttagcgtgatttatattttttttcgcctcgacatcat
ctgcccagatgcgaagttaagtgcgcagaaagtaatatcatgcgtcaatcgtatgtg
aatgctggtcgctatactgctgtcgattcgatactaacgccgccatccagtgtcgaa
aacgagctttcgagaacccttaatgcggccgcataacttcgtataatgtatgctata
cgaagttatGCATGAGTAGTTAGTTATCTTTTTGACAATGATCTCTTTTGAAAATAT
CTACTGTAGATTTGCATGGACGCACGTCGCCCATACGCCAAACTTTGGCAATGATAC
TCGTTATTCGTAATATCAGTCCGTCAAGGTGCTGTGATTTCTCTATTTTATATTGCC
TATTATTTTTTCAAATGATTTGAGCCGTTTTAAATTGA
